# Evaluation of tear osmolarity measured by I-Pen osmolarity system in patients with dry eye

**DOI:** 10.1038/s41598-021-87336-2

**Published:** 2021-04-08

**Authors:** Jongyeop Park, Youngjoo Choi, Gyule Han, Eunhae Shin, Jisang Han, Tae-Young Chung, Dong Hui Lim

**Affiliations:** 1 Samsung Medical Center, Department of Ophthalmology, Sungkyunkwan University School of Medicine, 81 Irwon-Ro, Gangnam-gu, Seoul, 06351 Republic of Korea; 2 Department of Ophthalmology, Dongguk University Gyeongju Hospital, Dongguk University School of Medicine, Gyeongju-si, Gyeongsangbuk-do Republic of Korea; 3 Department of Ophthalmology, Kangbuk Samsung Hospital, Sungkyunkwan University School of Medicine, Seoul, Republic of Korea; 4 Department of Health Sciences and Technology, Samsung Advanced Institute for Health Sciences and Technology, Sungkyunkwan University, Seoul, Republic of Korea

**Keywords:** Conjunctival diseases, Corneal diseases

## Abstract

This retrospective comparative study was to evaluate tear osmolarity measured by I-Pen osmolarity system (I-MED Pharma Inc, Dollard-des-Ormeaux, Quebec, Canada) in healthy subjects without dry eye disease (DED) and patients with DED, and its association with other ocular surface parameters. This study comprised 65 eyes of 65 patients. The ocular surface parameters including tear osmolarity with I-Pen osmometer of the patients who visited the refractive surgery center of Samsung Medical Center between January 1, 2020 and May 31, 2020 were retrospectively collected. The subjects were divided as asymptomatic normal group and symptomatic dry eye group. The distribution of tear osmolarity and its association with other ocular surface parameters were evaluated. Total thirty-two patients (32 eyes) were included in the control group, and 33 patients (33 eyes) were included in the DED group. Tear osmolarity was significantly higher in the DED group. Tear osmolarity was negatively correlated with tear break-up time, and the Schirmer test, and was positively correlated with Ocular Surface Disease Index symptom score. The cut-off value of 318 mOsm/L showed a sensitivity of 90.9% and specificity of 90.6% for diagnosing DED. The I-Pen osmometer can be considered suitable for use in the clinical setting, with good performance in DED diagnosis.

## Introduction

Dry eye disease (DED) is an inflammatory disorder that can lead to decreased visual quality, damage to the ocular surface, discomfort, and overall reduction in quality of life. The main features of DED are changes in tear film composition and the ocular surface inflammation that follows desiccating stress^1–4^. Tear film hyperosmolarity induces an inflammatory cascade at the ocular surface which initiates cytokine release, causing corneal epithelial damage and loss of goblet cells^1,2,5,6^. These lead to ocular discomfort and blurred vision in DED patients.

Based on this scientific evidence, tear osmolarity has been considered a global key pathogenic factor in DED patients^3^. The value of measuring tear osmolarity when diagnosing DED has been recognized and its potential to be the gold standard DED diagnostic test was proposed a long time ago. However technical problems hindered the use of this test in clinical practice. In 2012, a new tear osmometer, the Tearlab Osmolarity System (TearLab Corp, San Diego, CA, USA) was developed, which worked by measuring the electrical impedance of a 50-nL tear, collected from tear meniscus. The Tearlab osmometer has been shown to provide highly accurate results in the point-of-care setting with several studies showing the diagnostic performance of Tearlab osmometer in DED^7–10^.

More recently, a novel handheld osmolarity system, the I-Pen osmolarity system (I-MED Pharma Inc, Dollard-des-Ormeaux, Quebec, Canada), became commercially available and has been approved for sale in many countries^11^. The I-Pen osmometer measures tear osmolarity by measuring the electrical impedance of palpebral conjunctiva^12^. Previous in-vitro studies and in-vivo studies in normal healthy control without DED showed that measurements with both osmometer were different and that the measurements with I-Pen were significantly higher than those with Tearlab osmometer^13–15^. However, considering the difference in measurement site between the two osmometers (tear meniscus vs. palpebral conjunctiva), and the different operative mechanisms of the two devices, it is necessary to evaluate the clinical performance of I-Pen osmometer in DED patients^11,13^. The purpose of this study was to evaluate the potential usefulness of measuring tear osmolarity with an I-Pen osmometer in normal healthy controls and DED patients, and to investigate the correlation between tear osmolarity with the I-Pen and other subjective and objective parameters in DED.

## Materials and methods

### Subjects

This retrospective study was conducted in accordance with the tenets of the Declaration of Helsinki and was approved by the Ethical Committee of Samsung Medical Center (IRB no. 2020-06-122). IRB approved the exempt of informed consent, as the current study retrospectively collected the existing data, and the data was recorded in an anonymous manner such that subjects cannot be identified directly or through identifiers linked to the subject. A retrospective medical chart review was conducted on the patients who were tested with the I-Pen osmometer between January 1, 2020 and May 31, 2020 in Samsung Medical Center. All patients who visited the refractive surgery center of Samsung Medical Center for screening of refractive surgery candidates underwent full ophthalmic examination and ocular surface evaluation tear including tear osmolarity evaluation with I-Pen osmometer. The ocular surface evaluation was performed in the same order as follows: tear film osmolarity measurement with an I-Pen osmometer, tear film lipid layer thickness (LLT) measurement with a Lipiview interferometer (TearScience Inc, Morrisville, NC), non-invasive tear break-up time (NIBUT), tear meniscus height (TMH) measured with a Keratograph 5M (Oculus Optikgerate GmbH, Wetzlar, Germany), fluorescein tear break-up time (TBUT) measurement, corneal/conjunctival fluorescein staining scoring, and the anesthetized Schirmer test. After all the ocular examinations, each patient was asked to complete the Ocular Surface Disease Index (OSDI) questionnaire.

Among 74 patients who underwent ocular surface evaluation including osmolarity measured with I-Pen osmometer, 2 patients were excluded due to incomplete data. The subjects were divided as asymptomatic normal group and symptomatic dry eye group defined as follows. The normal healthy control group without dry eye was defined as having an OSDI symptom score ≤ 12, fluorescein tear-break up time ≥ 8 s, and Schirmer test ≥ 10 mm/5 min^16–18^. Subjects with symptomatic DED were defined as having an OSDI score ≥ 13 with fluorescein TBUT < 8 s or Schirmer test < 10 mm/5 min^17^. Only the data from either right or left eye of a subject which met the criteria was used. When both eyes met the criteria, only the data from right eyes were used for statistical analysis. Total thirty-two patients (32 eyes) were included in the control group, and 33 patients (33 eyes) were included in the DED group.

### Tear film osmolarity

The I-Pen osmometer was used to measure tear osmolarity. Measurement was always performed by the same investigator in the same examination room with a controlled temperature of 23.5–26.0 ℃ and humidity of 35–40%. A new disposable Single Use Sensor (SUS) was used for every measurement.

The SUS must first be inserted into the I-Pen device. Next, patients are asked to gently squeeze their eyelids closed for 30–60 s. Patients then open their eyes, and the tip of the SUS is placed at a 30°–45° angle directly onto the palpebral conjunctiva on the inside of the retracted lower eyelid, with gold node from the SUS in good contact with the palpebral conjunctiva. After a few seconds in this position, the handheld osmolarity system makes an audible beep and displays the osmolarity reading in milliosmole per liter on its LCD screen^14^. The I-Pen is known to measure the impedance of the extracellular fluid contained in the eyelid tissue^15^.

### Lipid layer thickness

Tear film lipid layer thickness (LLT) was measured by the LipiView Ocular Surface Interferometer. It projects white light over the lower third of the cornea, producing a color interference pattern as a result of specular reflection at the lipid-aqueous interface of the tear film^19^. Participants were instructed to blink freely throughout the imaging. A camera recorded a 20-s video of the tear film interference pattern and displayed the lipid layer thickness in “interferometric color units, ICU.” The average tear film LLTs were recorded. The Lipiview system had an upper cut-off of 100 ICU.

### Noninvasive tear break-up time (NIBUT) and tear meniscus height (TMH)

Noninvasive tear break-up time (NIBUT) was measured with a Keratograph 5M (Oculus Optikgerate GmbH, Wetzlar, Germany), which uses an infrared light source, to project a ring pattern from a placido disc onto the surface of the tear film^19^. Changes in image regularity are taken to indicate the onset of tear break up. The first disruption to the reflected mire image was detected by the instrument and recorded as the NIBUT. Also with the tear film scanning function of the Keratograph 5M, inferior tear meniscus images were captured and tear meniscus height (TMH) was measured as a straight line perpendicular to the lid margin at the central point relative to the pupil center using an integrated ruler^20^.

### Fluorescein tear break-up time (TBUT) and corneal-conjunctival staining score

A sterile fluorescein strip moistened with ocular irrigation solution was applied to the inferior fornix. Two minutes after the application of fluorescein, the patients were requested to blink several times to ensure adequate mixing of the dye and then keep their eyes open. The tear break-up time (TBUT) was examined under standard illumination using a slit-lamp microscope with cobalt-blue filter. TBUT is the time interval between the last blink and the appearance of the first dry spot on the corneal surfaces. The TBUT was measured three times with a stopwatch, and the mean value was recorded^8,20^. After the TBUT measurements, corneal and conjunctival staining was evaluated under a cobalt blue filtered light. The staining pattern was graded according to the NEI staining score^20^.

### Schirmer test

The type 1 Schirmer test with topical anesthesia was applied to measure aqueous tear production. Topical anesthetics (0.5% proparacaine hydrochloride; Paracaine, Hanmi, Seoul, Korea) were applied and a dry Schirmer test strip was inserted at the lower-lid margin at the junction of the middle and temporal third of both eyes, without touching the cornea or eye lashes. The patients were instructed to close their eyelids during the test for 5 min. After 5 min, the strip was removed and the amount of wetting was recorded.

### Ocular Surface Disease Index (OSDI)

Patients completed the standard questionnaires, the Ocular Surface Disease Index (OSDI) which is a structured instrument that quantify dry eye symptom severity. The OSDI questionnaire included 12 questions regarding dry eye symptoms during the past week; each symptom was graded from 0 to 4, for a final score of 0 (mild) to 100 (severe). A higher OSDI score represents greater disability.

### Statistical analysis

Descriptive statistical methods (mean ± standard deviation) were used to summarize the measurements by group. The independent *t*-test was used to compare groups on normally distributed parameters while the Mann–Whitney U test was used to compare groups on parameters which were not in normal distribution. To assess the relationships between parameters, Pearson correlation analysis was used for normally distributed variables, while Spearman correlation analysis was used for variables that were not normally distributed. All statistical analyses were conducted using SPSS (IBM SPSS Statistics for Windows, Version 22.0. Armonk, NY).

## Results

Ultimately, a total of 65 subjects were included in this study. The mean age was 26.54 ± 4.69 years, and 49.2% (32/65) were female. Thirty-two patients (32 eyes) were included in the control group, who showed an OSDI symptom score ≤ 12, fluorescein TBUT ≥ 8 s, and Schirmer test ≥ 10 mm/5 min. Thirty-three patients (33 eyes) who had an OSDI score ≥ 13 with either fluorescein TBUT < 8 s or Schirmer test < 10 mm/5 min were included in the DED group. Demographic data and dry eye parameters are described in Table 1. The mean tear osmolarity measured by the I-Pen was significantly higher in the DED group (331.33 ± 10.03) than in the control group (299.00 ± 16.46) (*p* < 0.001). The NIBUT was significantly lower in the DED group (6.27 ± 4.33 s) than in the control group (10.18 ± 5.12 s) (*p* = 0.035). And the fluorescein TBUT was significantly lower in the DED group (4.56 ± 2.08 s) than in the control group (11.84 ± 3.99 s) (*p* = 0.001). Corneal staining and conjunctival staining were significantly higher in the DED group (1.96 ± 1.94 and 2.39 ± 3.41, respectively) than in the control group (0.96 ± 1.23 and 0.93 ± 1.33, respectively) (*p* = 0.021 and *p* = 0.035, respectively). Schirmer score was significantly lower in the DED group (11.84 ± 3.99 mm) than in the control group (17.82 ± 6.17 mm) (*p* < 0.001, respectively). The OSDI score was significantly higher in the DED group (30.92 ± 11.36) compared to the control group (7.55 ± 4.79) (*p* < 0.001).

**Table 1:** Baseline characteristics and dry eye parameters in the control and DED groups.

	Control group (n = 32)	DED group (n = 33)	*p*-value
Age (years)	26.18 ± 3.58	26.95 ± 5.74	0.562
Sex (female, %)	17 (53.1%)	15 (45.5%)	0.863
Osmolarity	299.00 ± 16.46	331.33 ± 10.03	** < 0.001**
LLT	89.70 ± 57.627	80.15 ± 18.79	0.370
NIBUT	10.18 ± 5.12	6.27 ± 4.33	**0.035**
TMH	0.22 ± 0.05	0.18 ± 0.06	0.070
TBUT	11.84 ± 3.99	4.56 ± 2.08	**0.001**
Corneal staining	0.96 ± 1.23	1.96 ± 1.94	**0.021**
Conjunctival staining	0.93 ± 1.33	2.39 ± 3.41	**0.035**
Schirmer test	17.82 ± 6.17	5.30 ± 2.17	** < 0.001**
OSDI Score	7.55 ± 4.79	30.92 ± 11.36	** < 0.001**

Bold values indicate statistical significance (*p* < 0.05).

LLT, lipid layer thickness; NIBUT, non-invasive tear break-up time; TMH, tear meniscus height; TBUT, tear break-up time; OSDI, ocular surface disease index.

**Figure 1:**
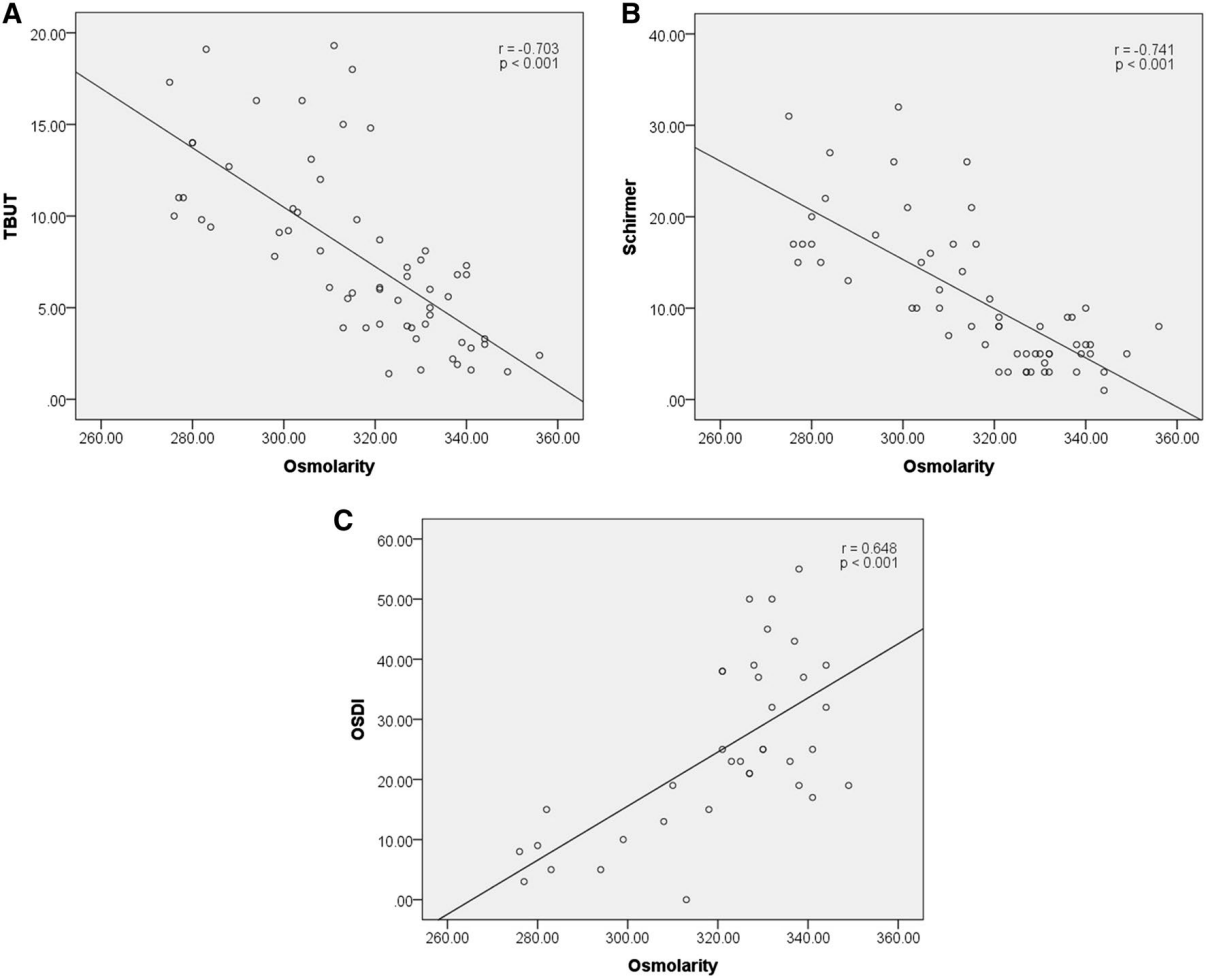
Correlation between tear osmolarity and other dry eye parameters (Tear break-up time (TBUT), Schirmer test, Ocular Surface Disease Index (OSDI) score). (**A**) negative correlation between TBUT and osmolarity; (**B**) negative correlation between Schirmer test and osmolarity; (**C**) positive correlation between OSDI and osmolarity.

**Figure 2:**
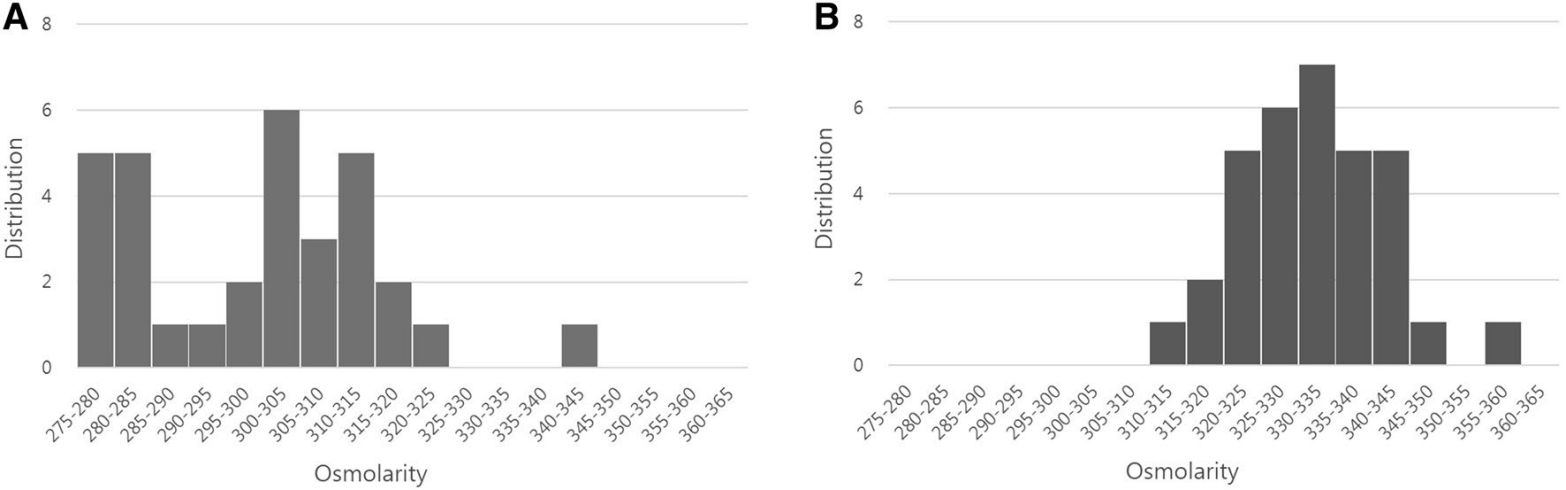
Distribution of osmolarity measured with the I-Pen osmometer. (**A**) distribution of osmolarity in the non-dry eye control group; (**B**) distribution of osmolarity in the dry eye disease group.

**Figure 3:**
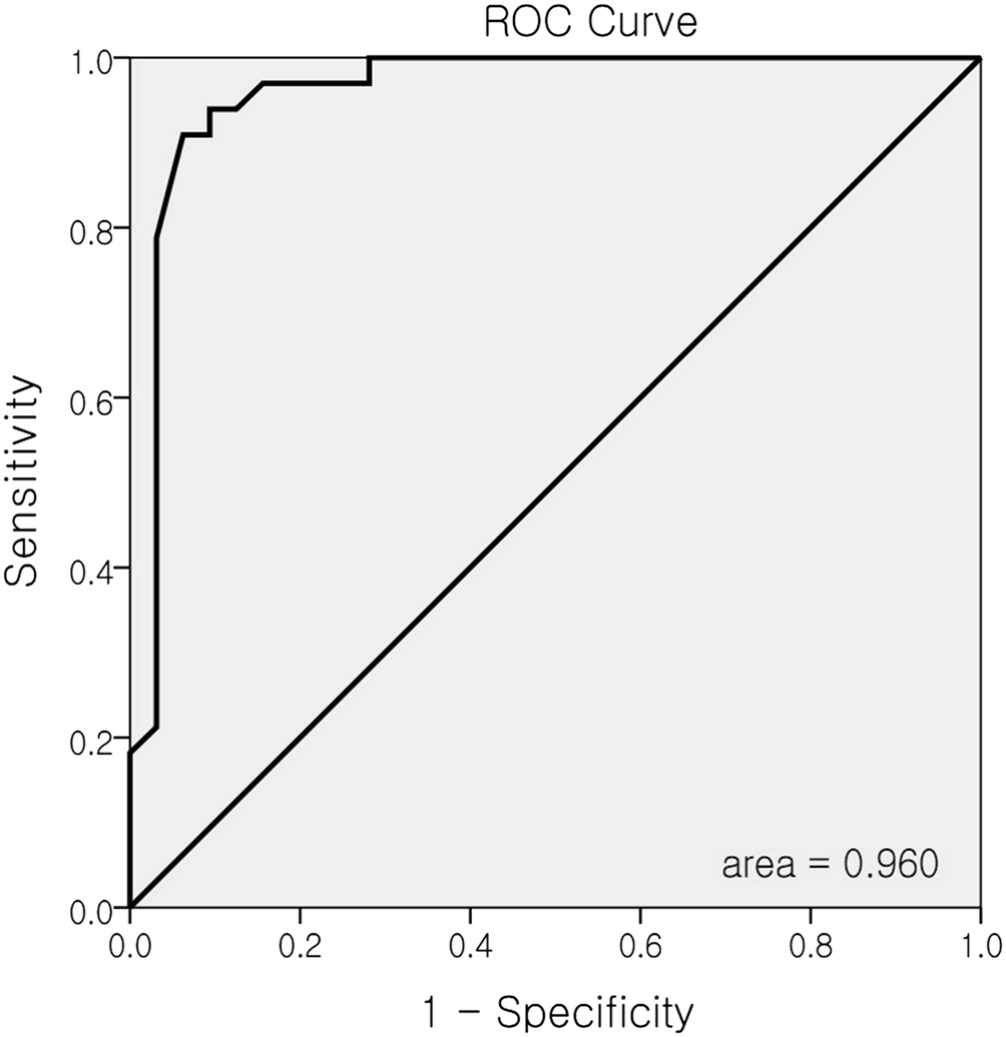
Receiver operating characteristic (ROC) curve analysis resulted in 318 mOsm/L as the threshold to classify dry eye disease. Sensitivity = 90.9%, Specificity = 90.6%.

The correlations between tear osmolarity and other dry eye parameters are shown in Table 2. There was a negative correlation between TBUT and osmolarity (r =  − 0.703, r^2^ = 0.494; *p* < 0.001) (Fig. 1A) and between the Schirmer test and osmolarity (r =  − 0.741, r^2^ = 0.549; *p* < 0.001) (Fig. 1B). There was a moderate positive correlation between OSDI and osmolarity (r = 0.648, r^2^ = 0.419; *p* < 0.001) (Fig. 1C).The distribution of osmolarity measurements using the I-Pen in both the control and DED groups is shown in Fig. 2A and Fig. 2B. The median value was 302 mOsm/L in the control group (Fig. 2A), and 330 mOsm/L in the DED group (Fig. 2B). The cut-off value of 318 mOsm/L showed the greatest area under the receiver operating characteristic (ROC) curve. The sensitivity of identifying patients with clinically significant DED was 90.9% with a specificity of 90.6% when the threshold was 318 mOsm/L (Fig. 3).

**Table 2:** Relationship between tear osmolarity and other dry eye parameters.

	Tear osmolarity (mOsm/L)	
N = 65	R	*p*-value
LLT	− 0.022	0.864
NIBUT	− 0.347	0.070
TMH	− 0.332	0.079
TBUT	− 0.703	** < 0.001**
Corneal staining	0.195	0.136
Conjunctival staining	0.223	0.083
Schirmer test	− 0.741	** < 0.001**
OSDI Score	0.648	** < 0.001**

Bold values indicate statistical significance (*p* < 0.05).

## Discussion

In this study, patients were classified into asymptomatic normal control or symptomatic dry eye groups and the relationship between tear osmolarity measured with an I-Pen osmometer and recent non-invasive dry eye tests (LLT, NIBUT, and TMH), traditional diagnostic tests (Schirmer test, TBUT, and ocular surface staining) and patient symptoms was evaluated. We found that tear osmolarity was significantly higher in the DED group with a median value of 330 mOsm/L and tear osmolarity as measured by an I-Pen was significantly correlated with TBUT, Schirmer score, and OSDI symptom score.

Previous studies utilized the Tearlab osmometer, which was commercialized in 2012. The TearLab consists of collection pen for tear collection, a reader into which the collection pen is docked and single-use disposable Test Cards^21^. The single-use Test Card collects 50 nL of tear fluid from lower tear meniscus by passive capillary action. The reader uses a temperature compensated impedance to measure tear osmolarity on the collected tear fluid sample. The Tearlab has been regarded as the gold standard for measuring tear osmolarity, with the cut-off value of 308 mOsm/L for the diagnosis of DED^13^.

The I-Pen osmometer became available more recently. The I-Pen determines tear osmolarity by measuring electrical impedance, similar to the Tearlab osmometer, but of the saline concentration of the extracellular fluid contained in conjunctival tissues as opposed to the tears themselves^12^. The values measured by both osmometers are not exactly the same, and measurements by the I-Pen are known to be higher than those measured by Tearlab^14,15^. The manufacturer’s instruction manual provides reference data on tear osmolarity measured by I-Pen including a mean value of 300 mOsm/L and range of 275–316 mOsm/L for normal patients, and a mean value of 327 mOsm/L for dry eye patients^12,22^. As the Tearlab and I-Pen are not giving results that are directly comparable, the cut-off value of Tearlab cannot be used with the I-Pen, and unique values should be calculated for the I-Pen^13^.

In this study, tear osmolarity measured by the I-Pen osmometer was significantly different between subjects without or with DED, with the values similar to the manufacturer’s manual^12^. When adopting the cut-off value of 318 mOsm/L, the I-Pen differentiated 90.9% of DED patients (sensitivity) and 90.6% of normal subjects (specificity). Though both osmometers were not directly compared in this study, the clinical performance of the I-Pen might be comparable to that of the Tearlab, which showed 90.0–92.0% for diagnosing normal subjects without DED^2,10^. Tear osmolarity can be affected by a number of environmental factors, including temperature, humidity, air flow, patient general condition, systemic medication, and seasonal or diurnal variation^23^. Further investigations should be conducted to establish a cut-off value for the I-Pen osmometer, that considers those factors.

In this study, tear osmolarity measured with the I-Pen was significantly correlated with TBUT, the Schirmer test, and the OSDI. This result affirms the current pathogenesis of dry eye as decreased tear production and increased evaporation rate that preceed findings of changes in tear osmolarity. According to the Dry Eye Workshop (DEWS), DED results from any conditions in which lacrimal secretion decreases or tear evaporation increases include aqueous deficient dry eye (ADDE) and evaporative dry eye (EDE)^3,4^. The possible mechanisms reported by the DEWS, suggest that decreased lacrimal gland secretion, as in the case of Sjogren’s syndrome, is the main cause of ADDE, and several intrinsic and extrinsic conditions cause EDE, with the most predominant causes being the diseases that affect the lid margin meibomian gland. Other common etiologies include intrinsic dysfunction, i.e., low blink rate, and extrinsic problems, i.e., vitamin A deficiency and ocular allergies. The Schirmer test which assesses tear production, and TBUT that reflects tear evaporation and tear film instability are the most vital clinical signs for DED, which were found to be correlated with tear osmolarity in this study.

The OSDI questionnaire is based on questions including ocular discomfort, visual symptoms, and environmental triggers, and its validity in diagnosing dry eye and discriminating severity grade has been established^24^. Using tear osmolarity to diagnose DED in patients who present with typical dry eye symptoms with ocular surface staining is not usually necessary^25^. However, tear osmolarity may play a role in establishing the objective metrics that are associated with subjective discomfort^25^.

These findings are consistent with other studies. Suzuki et al. reported a negative correlation between tear osmolarity and the Schirmer test and TBUT in their cohort of dry eye patients, which included both ADDE and EDE patients^25^. Utine et al. found a positive correlation between tear osmolarity and OSDI score and a negative correlation between tear osmolarity and both the Schirmer test and TBUT^26^. Ozulken et al. reported that tear osmolarity was strongly and inversely correlated with both TBUT and NIBUT^27^. As the previous studies all used the Tearlab osmometer, a direct comparison with the I-Pen osmometer is not technically possible. However, it is known that tear osmolarity is very valuable for the diagnosis and treatment of DED, regardless of the specific osmometer used to make the diagnosis^7,13^.

To the best of our knowledge, just one research has ever reported the tear osmolarity measured by I-Pen in DED patients. Shimazaki et al., adopted the I-Pen osmometer in Japanese DED patients and reported its clinical utility^28^. In the study, symptomatic eyes with TBUT ≤ 5 s, were regarded as having DED, and noneligible subjects were defined as normal control, irrespective of Schirmer test or ocular surface staining. Such definition of DED is known as ‘short TBUT-type dry eye’, with higher prevalence in Japan^29^. They reported that tear osmolarity did not differ between eyes with and without short TBUT-type DED. In addition, they found no correlation between osmolarity values obtained with the I-Pen and subjective symptom score or other objective DED parameters. Such discrepancy between our results and those of Shimazaki et al., can be explained by the different definition of DED and difference in prevalence of short TBUT-type DED. Also, as they admitted, operator error resulting in ocular irritation and lacrimation, leading to inaccuracy in measuring tear osmolarity is possible due to the multiple examiners in their multicenter study design^28^.

There are certain limitations to this study. The sample size was relatively small. There was one outlier in the control group, which showed tear osmolarity of 340 mOsm by I-Pen osmometer. The subject showed an OSDI of 6, TBUT of 11 s, Schirmer of 15 mm/5 min and no corneal staining. The outlying data might be due to the measurement error of I-Pen. However, considering the relatively small sample size, the outlying data was not handled. Further study performing the repeated measurements with the I-pen in a larger population might reveal the measurement variability of I-pen osmometer. Also, the dry eye severity grade distribution among the study patients was not considered. The majority of the patients were rated as dry eye severity grade 2 according to the DEWS grading. Considering that tear osmolarity might be an indicator disease severity, this is a limitation of this study. A subsequent, prospective study that compares I-Pen osmolarity with DED severity grading would provide a better understanding of I-Pen osmolarity in DED patients. Lastly, the mean age of the subjects in this study was relatively young. However, age is not known to be a confounding factor when measuring tear osmolarity^30^. Considering that the prevalence of DED increases with age, further studies including a broader age range are necessary.

This is the first study to determine the association between tear osmolarity as measured by the I-Pen and dry eye parameters. The tear osmolarity acquired by the I-Pen osmometer was significantly higher in patients with dry eye compared to non-dry eye controls, and correlated negatively with TBUT and the Schirmer test, and positively with OSDI symptom score. Although tear osmolarity cannot be used as the sole indicator of DED, it seems to be a helpful parameter for screening and diagnosing DED.
